# Blurred Lines: Pathogens, Commensals, and the Healthy Gut

**DOI:** 10.3389/fvets.2015.00040

**Published:** 2015-10-01

**Authors:** Paul Wigley

**Affiliations:** ^1^Institute for Infection and Global Health, University of Liverpool, Liverpool, UK

**Keywords:** chicken, microbiome, gut health, probiotics, commensal, *Campylobacter*, *Clostridium perfringens*, *Escherichia coli*

## The Chicken Microbiome and Health

Detailed studies of the chicken microbiome have emerged in recent years, largely due to the impact of next-generation sequencing (NGS). We increasingly understand how the microbiome is important in health, in development of the gut and the immune system, and in maintenance of homeostasis. Manipulation of the microbiota directly through probiotics or antimicrobials or indirectly through feed and feed additives has long been used by the poultry industry to increase growth rates and feed conversion, to improve gut health, and to reduce the burden of pathogens and, in particular, to reduce the load of foodborne zoonotic pathogens such as *Salmonella* and *Campylobacter*. We can now begin to mechanistically determine how these treatments affect the microbiota and the wider host, and this understanding will allow us to use more targeted approaches in the future. In terms of food security, increasing yield is clearly a good thing. However, it is far from clear what represents a “healthy” microbiome, and the lines between what is a “harmless” commensal and what is a pathogen are often blurred. As such an understanding of the microbial ecology of the gut and how this is affected by manipulation of the microbiome or indeed treatment of “pathogens” is essential in ensuring that treatments intended to improve health and productivity do not in fact cause more problems.

## Is it a Pathogen or a Commensal?

The chicken microbiome consists of around 1,000 bacterial species, though the composition varies over time, between breeds and lines of birds, between flocks, individuals, and at different sites within the gut (1–5). Proteobacteria make up a relatively small amount of species in the microbiome, but among these species are a number that may cause disease in the chicken, notably *Escherichia coli* and *Clostridium perfringens*, and as such are often considered pathogens (4–6). In contrast, the foodborne zoonotic pathogen *Campylobacter jejuni* is also found frequently as a component of the cecal microbiome but is often considered to be a “harmless commensal.” However, in reality, these species can have the properties of either pathogen or commensal depending on the bacterial pathotype, host immune status, diet, and coinfection.

Of these three exemplars, *E. coli* has perhaps the least direct impact on gut health. However, extraintestinal infections are a considerable health problem in both broiler and layer chicken production. Isolates associated with disease are termed avian pathogenic *E. coli* or APEC. Much effort has been directed at understanding the virulence factors and pathogenesis of APEC, and there are clearly a number of pathotypes that can cause disease (7, 8). However, wider analysis of isolates associated with systemic infection or colibacillosis of broiler chickens and those associated with a healthy gut show that disease may be caused by isolates that harbor few, if any, APEC-associated virulence factors while apparently “commensal” isolates carry numerous virulence factors (9). The implication is that in many clinical cases of colibacillosis, commensal bacteria act as an opportunistic pathogen due to host factors, environmental stress, poor management, or as a secondary infection. As such infections are rarely investigated in detail such as genotyping of isolates; the more generic term of APEC has become associated with all *E. coli* isolated from diseased chickens rather than those *E. coli* isolates that are primary pathogens *per se*.

*Campylobacter jejuni* is the most common cause of foodborne human gastrointestinal infection worldwide. Chicken is the main reservoir of infection with around 70% of UK retail chicken contaminated in recent surveys (10, 11). *C. jejuni* colonizes the lower gastrointestinal tract of the chicken to a high level and has been considered to be a commensal due to the absence of clinical disease in experimental infection studies (12). However, in recent years, we have begun to reassess this paradigm. Experimental infection of broiler breeds with *C. jejuni* leads to an inflammatory response and changes to gut structure (13–16). Generally, it would appear that this inflammation is regulated by IL-10-producing cells, but in some broiler breeds, regulation appears to be dysfunctional and infection may lead to prolonged inflammation, damage, and diarrhea. Does this mean that *C. jejuni* is truly a pathogen of the chicken or more a reflection of dysregulation of mucosal immune regulation? Indeed, poor gut health is often considered as a problem for broiler chickens. Wet litter, due to loose feces mixed with the bedding substrate, and dysbacteriosis are frequent problems in broiler production that affect productivity and animal welfare both directly and through resulting problems such as pododermatitis and hock burn (17–19). Modern broiler chickens have been successfully bred to efficiently convert grain into protein and grow rapidly, reaching slaughter weight at 6–7 weeks of age and we increasingly understand the genetic basis for this (20, 21). This, however, may have consequences; well-documented musculoskeletal problems are being addressed, but problems with gut health may be less obvious and harder to deal with. One may pose the question to what extent are these problems related to the composition of the microbiota and development of a healthy gut or more a consequence of a defect in their gut physiology or immune function? Additionally, to what extent could inappropriate or poorly regulated responses to the “normal” microbiota be contributing to poor gut health? The example of *C. jejuni* illustrates how the balance in maintaining a healthy gut is likely to be influenced by a large number of both host and microbial factors.

Clostridia are a major component of the proteobacteria in the chicken microbiome (5). Of these species, *C. perfringens* is the most important in poultry health. Variants of *C. perfringens* are associated with the gut of many species, and it can be generally considered as a commensal. Yet, it may produce toxins associated with disease including human food poisoning or in necrotic infections of the gut or deep tissue. In the chicken, the *C. perfringens* toxin group A has become most associated with necrotic enteritis (NE), these isolates producing alpha and particularly netB toxins (22). Despite *C. perfringens* producing these toxins being closely associated with NE, it had proved very difficult to fulfill Koch’s postulates as such isolates are frequently found in healthy birds and reliable experimental infection models for NE based on *C. perfringens* infection alone have proved hard to develop. This is largely due to most clinical disease being multifactorial involving predisposing factors such as coinfection particularly with species of the apicomplexan protozoan parasite *Eimeria* or due to dietary factors such as diets high in non-starch polysaccharides (NSPs; wheat, rye, and barley) or animal proteins that provide favorable conditions for the growth of *C. perfringens* A and stress on birds in production (23). Again it is difficult to define *C. perfringens* as a true gut pathogen, but more of an opportunist that frequently makes up part of the microbiome.

## Manipulation of the Microbiome: Past and Future Implications for Gut Health

Historically, currently and likely into the future, the chicken microbiome has been manipulated perhaps more than any other vertebrate species through the use of growth-promoting antimicrobials, prebiotic and probiotic treatments, and dietary additives (24–27). Feed additives such as enzymes have been used to increase productivity. For example, the use of phytases to allow the breakdown of plant phytates to be utilized in diet (28). Other additives such as plantain NSPs have been proposed to reduce the burden of infections such as *Salmonella* (29). The use of growth-promoting antimicrobials has been banned in the European Union since 2006 and their use in the USA is under increasing pressure due to their role in development of antimicrobial resistance. Not unexpectedly, the use of antimicrobial growth promoters affects the composition of the microbiota (30, 31), and equally the withdrawal of both growth-promoting and anticoccidial drugs will lead to change in the microbiota composition in commercial flocks. Interestingly, a recent study on drug-free broiler production systems in Canada showed an increase in *C. perfringens* (32). Anecdotally, the increased prevalence of both NE and colibacillosis in Europe has been blamed, at least in part, on the withdrawal of growth promoters. While the overriding problems associated with the emergence of antimicrobial resistance rightly mean that growth-promoting antibiotics have been or are being withdrawn, it clearly illustrates how the manipulation of microbiota can have positive effects on health of the chicken. Equally, we need to be aware that changing the microbiota or modulating host responses that are affected by or effect changes upon the microbiome may have undesirable effects. In the case of growth promoters, this was their role in the development of resistant bacteria and potential drug residues in the food chain. As such a better understanding of microbial ecology and how interventions impact on the microbiota and the host is needed before we adopt such changes wholesale. Manipulation of the microbiome may be used to improve productivity, although the consequences of removing “detrimental” or enriching “beneficial” taxa are likely to go beyond improving feed conversion. A perturbed microbiome may reveal commensals as having pathogenic potential and lead to problems in development of the gut and immune system and poor gut homeostasis. Manipulation of the gut, the microbiome, and the immune response has all been proposed in reducing the burden of carriage of foodborne bacterial pathogens. Our work on feed supplementation with plantain NSP showed successful inhibition of *Salmonella* invasion (29), but rather unexpectedly lead to increased colonization of the intestinal tract with *Salmonella gallinarum*. *S. gallinarum* has evolved with several defective metabolic pathways that make it a poor colonizer of the chicken gut, but supplementation with plantain NSP increased colonization either through a direct nutritional source or more likely utilization of breakdown products of microbiota components (33). Equally immunological manipulation may have unexpected consequences. Both colonization of *Salmonella* and *Campylobacter* are accompanied by regulation of innate responses to these bacteria in the gut (13, 34, 35). It has been proposed that depletion of the regulatory CD4^+^ CD25^+^ T-cell population will enhance clearance and thereby reduce the public health risk due to these pathogens (36, 37). A downside of this may be increased inflammation and more significantly loss of regulation to components of the microbiome, again blurring lines between pathogen and commensal and leading to poor gut and poor health.

## Conclusion

Ultimately, the “take-home” message in this article is that the power of NGS and metagenomic approaches allow us to understand the composition of microbiomes in multiple individuals of a livestock species quickly and relatively easily. We can associate individual taxa and species with good or poor outcomes in productivity or health. Yet, this power needs to be tempered with (often substantial) gaps in our understanding of microbial ecology within the gut. Can changing the microbiota lead to perturbation of gut regulation? As we have seen, there are blurred lines between pathogens and commensals, and so can changes to remove apparent pathogens have negative consequences on other aspects of gut health or could the promotion of “good bacteria” lead to emergence of “new pathogens.” The historical example of growth-promoting antimicrobials illustrates the point. Their use was successful in increasing productivity yet almost certainly has contributed to antimicrobial resistance (38). Their subsequent withdrawal is now resulting in problems in our faster growing modern broiler chickens. Our understanding of the microbiome and its manipulation offers a wealth of opportunities, though may not be without risk.

## Conflict of Interest Statement

The author declares that the research was conducted in the absence of any commercial or financial relationships that could be construed as a potential conflict of interest.

## Funding

This work was supported by the Biotechnology and Biological Science Research Council though grant number BB/J017353/1.
